# 7,8-Dihydroxyflavone improves neuropathological changes in the brain of Tg26 mice, a model for HIV-associated neurocognitive disorder

**DOI:** 10.1038/s41598-021-97220-8

**Published:** 2021-09-16

**Authors:** Joseph Bryant, Sanketh Andhavarapu, Christopher Bever, Poornachander Guda, Akhil Katuri, Udit Gupta, Muhammed Arvas, Girma Asemu, Alonso Heredia, Volodymyr Gerzanich, J. Marc Simard, Tapas Kumar Makar

**Affiliations:** 1 Institute of Human Virology, Baltimore, MD 21201 USA; 2 Research Service, Veterans Affairs Center, Baltimore, MD 21201 USA; 3 Department of Neurosurgery, University of Maryland, Baltimore, MD 21201 USA

**Keywords:** Cell biology, Neuroscience

## Abstract

The combined antiretroviral therapy era has significantly increased the lifespan of people with HIV (PWH), turning a fatal disease to a chronic one. However, this lower but persistent level of HIV infection increases the susceptibility of HIV-associated neurocognitive disorder (HAND). Therefore, research is currently seeking improved treatment for this complication of HIV. In PWH, low levels of brain derived neurotrophic factor (BDNF) has been associated with worse neurocognitive impairment. Hence, BDNF administration has been gaining relevance as a possible adjunct therapy for HAND. However, systemic administration of BDNF is impractical because of poor pharmacological profile. Therefore, we investigated the neuroprotective effects of BDNF-mimicking 7,8 dihydroxyflavone (DHF), a bioactive high-affinity TrkB agonist, in the memory-involved hippocampus and brain cortex of Tg26 mice, a murine model for HAND. In these brain regions, we observed astrogliosis, increased expression of chemokine HIV-1 coreceptors CXCR4 and CCR5, neuroinflammation, and mitochondrial damage. Hippocampi and cortices of DHF treated mice exhibited a reversal of these pathological changes, suggesting the therapeutic potential of DHF in HAND. Moreover, our data indicates that DHF increases the phosphorylation of TrkB, providing new insights about the role of the TrkB–Akt–NFkB signaling pathway in mediating these pathological hallmarks. These findings guide future research as DHF shows promise as a TrkB agonist treatment for HAND patients in adjunction to the current antiviral therapies.

## Introduction

The introduction of combined antiretroviral therapy (cART) significantly increased the lifespan of people with HIV (PWH), turning a fatal disease into a chronic one^1^. Despite the increased life expectancy, however, the quality of life of these patients has decreased due to the numerous comorbidities associated with aging. The low but persistent level of HIV infection in the cART-treated PWH population has increased the susceptibility of HIV-associated neurocognitive disorder (HAND). HAND collectively describes the cognitive impairment that results from neurologic and central nervous system (CNS) involvement of HIV-1^2^, which currently affects over 50% of the PWH population^3^. Neurocognitive dysfunctions caused by HIV-1 can present as minor cognitive deficits or severe dementia and can be seen in both the earliest and latest stages of infection^4^. HIV-1 is known not to infect neurons, but certain cellular and subcellular alterations such as neuronal cell loss, dendritic simplification, and decreased synaptic density are thought to be possible causes of behavioral changes^5^. Furthermore, residential cells such as macrophages, microglia, and astrocytes are a primary target of HIV-1 infection^6,7^. This neuropathology may be due in part to impaired mitochondrial biogenesis and dynamics^8^ and neuroinflammation^4,9^.

Brain derived neurotrophic factor (BDNF), a member of the neurotrophin family, is a ligand of the tropomyosin-receptor-kinase B (TrkB) receptor and is essential in maintaining neuronal modulation of dendritic branching and spines in the cortex and long-term potentiation in the hippocampus^10,11^. Prominent in the CNS, signaling via the BDNF-TrkB pathway is known to play a role in learning, memory and the preservation of cortical circuits by mediating synaptic plasticity^12^. BDNF has been studied to show its possible protective effects in neuroAIDS models^13^. Studies in animal models have shown that BDNF plays an ameliorative and neuroprotective role in degenerating neurons in HIV-associated dementia (HAD)^14^. Clinically, the expression of BDNF is down-regulated in PWH compared to normal individuals without HIV-1^15^. Additionally, PWH with dementia exhibit even lower levels of BDNF in the cerebrospinal fluid^16^. In both rodents and humans, lower levels of BDNF have been implicated in the loss of hippocampal and cortical synapses and impairments in spatial learning and memory^11^. Therefore, BDNF could be a potential therapeutic agent for the treatment of diseases characterized by loss of synaptic plasticity such as HAND.

Additionally, studies that suggest ART therapy can be neurotoxic and affect BDNF expression. For example, HIV drug nevirapine has been shown to reduce BDNF levels^17,18^. However, administration of BDNF is limited due to its brief half-life, large molecular size, and poor blood–brain barrier (BBB) penetration^19^. Hence, an alternative molecule which mimics BDNF function and is also easily administered is optimal.

Flavonoids have been proposed as mimicking agents due to the aforementioned properties that BDNF lacks^20^. 7,8-dihydroxyflavone (DHF) is an orally bioavailable small molecule that has recently been identified as a high affinity TrkB agonist. DHF has the ability to cross the BBB and bind to the TrkB receptor, potentially mimicking the neuroprotective effects of BDNF^21^. Its effects have been shown in various other neurological diseases^22–25^. However, it has not yet been determined whether this compound exhibits neuroprotective properties in HAND.

The HIV-1 Tg26 mouse model is representative of the long-term effects induced by viral proteins on the host and clinically relevant to ART-controlled PWH^26^. Recent studies conducted by Putatunda et al., 2018 demonstrated that compared to wild-type (WT) mice, HIV-1 Tg26 mice have neurological deficits both in the early and late stages. Tg26 mice experience deficits in neuronal differentiation during adult neurogenesis. Middle-aged HIV-1 Tg26 (+ / −) mice also exhibit deficits in spatial memory acquisition, short-term memory retention, and long-term retention^27^. Therefore, Tg26 mice expressing HIV-1 genes in the brain share several pathological hallmarks with HAND and are an appropriate animal model.

Our aim in the present study was to investigate the neuroprotective effects of DHF in the hippocampus and cortex of the brains of Tg26 mice, a murine model for HAND, with a focus on of HIV-1 chemokine coreceptors C-X-C chemokine receptor type 4 (CXCR4)/C–C chemokine receptor type 5 (CCR5) expression, inflammatory activity, mitochondrial damage, and associated signaling mechanisms. We chose to look at these specific regions because they are integral for memory and cognitive function, which are impaired during HAND^2^. Previously, it has been shown that the hippocampus selectively has relatively high viral loads^28^. In the brains of Tg26 mice, we observed astrogliosis, increased expression CXCR4 and CCR5, neuroinflammation, and mitochondrial damage. Hippocampi and cortices of DHF treated mice exhibited a reversal of these pathological changes. DHF successfully increased the phosphorylation of TrkB, providing new insights about the role of the TrkB–Akt–F–kB signaling pathway in mediating these pathological hallmarks.

## Materials and methods

### Animals

HIV-1 transgenic Tg26 mice FVB/N expressing high levels of 7 of the 9 HIV-1 proteins were established using the 7.4 kb transgene construct lacking the 3 kb sequence overlapping the *gag/pol* region of provirus pNL4-3 as described previously^29^. The model was originally obtained from the National Institute of Dental Research, and the colony has been maintained in the Institute of Human Virology (IHV) since 1995 by cross breeding heterozygous mice and continuously backcrossing to the wild-type FVB/N. To ensure little to no genetic drift, we also obtained colonies from Jackson Labs for comparison.

Wild-type (WT) mice with an FVB/N genetic background generated from the same litter of Tg26 mice were used as controls for these studies. Female transgenic (Tg26) mice were housed under pathogen-free conditions at the animal facility of the Institute of Human Virology, University of Maryland School of Medicine, Baltimore. Female mice were used because Tg26 female mice show more cognitive deficits including short and long term spatial memory, loss in novel object location memory, and learning deficits, while male Tg26 mice don’t express any such changes^30^. The study was carried out in compliance with the ARRIVE guidelines. All experimental procedures were conducted following NIH guidelines under an Institutional Animal Care and Use Committee-approved protocol from the University of Maryland School Of Medicine, Baltimore.

### Drug

We administered DHF (Tokyo Chemical Industry) intraperitoneally at a dose of 5 mg/kg in a 200 μl vehicle of 0.2% dimethyl sulfoxide (DMSO) in phosphate-buffered saline (PBS). Mice were divided into 3 groups untreated-Tg26 mice and DHF-treated Tg26 mice (Tg + DHF) and Wild type mice. Tg26 + DHF mice received a daily dose from day 90 to day 120. Vehicle-treated Tg26 mice received a daily dose of vehicle (200 μl of 0.2% DMSO in PBS) from day 90 to day 120.

### Tissue pathology

Mice were euthanized on day 120 using the general anesthetic isoflurane. Brains were removed for analysis. Paraffin sections of the brain were prepared as previously described^25^. 7 μm thick sections were stained.

### Immunohistochemistry

Immunohistochemistry was performed as previously described^25^ using VECTASTAIN ABC kits (Vector Laboratories, Burlingame, CA, USA). Primary antibodies used in the experiment are listed in Table 1. Nuclei were counterstained with hematoxylin. Slides were examined using standard bright field microscopy.

**Table 1:** List of primary antibodies.

Antibody	Target	Vendor	Concentration
CXCR4	C-X-C Chemokine Receptor Type 4	Abcam, Cambridge, MA, USA	1:20 (IHC)
CCR5	C–C Chemokine Receptor Type 5	Abcam, Cambridge, MA, US	1:20 (IHC)
Anti GFAP	Glial fibrillary acidic protein	EMD Millipore, Billerica, MA, USA	1:100 (IHC)
Anti P-TRKB	Phospho-tropomyosin receptor kinase B	Abcam, Cambridge, MA, USA	1:500 (IHC)
Anti P-AKT	Phospho-protein kinase B	Cell Signaling Technology, Boston, MA, USA	1:50 (IHC)
Anti IFN-y	Proinflammatory cytokine	Bioss Antibodies, Boston, MA, USA	1:100 (IHC)
Anti TNF-a	Pro-inflammatory cytokine	Santa Cruz Biotechnology, Santa Cruz, CA, USA	1:500 (IHC)
Anti IL-10	Anti-inflammatory cytokine	Abcam, Cambridge, MA, USA	1:400 (IHC)
Anti NFkB	Nuclear factor kappa-light-chain-enhancer of activated B cells	Cell Signaling Technology, Boston, MA, USA	1:200 (IHC)
Anti TLR4	Toll-like receptor 4	Novus Biotechne	1:200 (IHC)
Anti Citrate Synthase	Enzyme marker for intact mitochondria	Genetex, Irvine, CA, USA	1.500 (IHC)
Anti SIRT3	Mitochondrial protein	Cell Signaling Technology, Boston, MA, USA	1:100 (IHC)
Anti PGC1-a	Peroxisome proliferator-activated receptor gamma coactivator 1 α	Abcam, Cambridge, MA, USA	1:100 (IHC)
Anti MFN-2	Mitofusin 2	Genetex, Irvine, CA, USA	1:500 (IHC)
Anti FIS-1	Mitochondrial fission 1 protein	Genetex, Irvine, CA, USA	1:200 (IHC)
Anti PACS2	Phosphofurin Acidic Cluster Sorting Protein 2	Invitrogen	1:500 (IHC)
Anti VDAC-1	Voltage Dependent Anion Channel 1	Abcam, Cambridge, MA, USA	1:500 (IHC)

### Analysis of histological images using ImageJ

Hippocampal and cortical regions of the brain sections were selected from all mice for pathology and immunohistochemistry. Histological quantification was performed by a blind observer using Image J. All cell labeling experiments (antibodies listed in Table 1) were quantified based on the number of positive cells/field. (Each field = 400 × magnification picture). All fields covering the hippocampus and cortex were analyzed from each brain. Antibodies are listed below.

### Statistical analysis

Statistical analyses were done using Prism software (GraphPad, San Diego, CA). Values are expressed as means ± SEM. Statistical analysis was performed with one-way ANOVA, and Bonferroni's Multiple Comparison Post Hoc Test was used for determining statistical significance between each group. Statistical significance was accepted at the 95% confidence level (*p* < 0.05).

## Results

### DHF treatment induces phosphorylation of TrkB and Akt in Tg26 hippocampus

**Figure 1:**
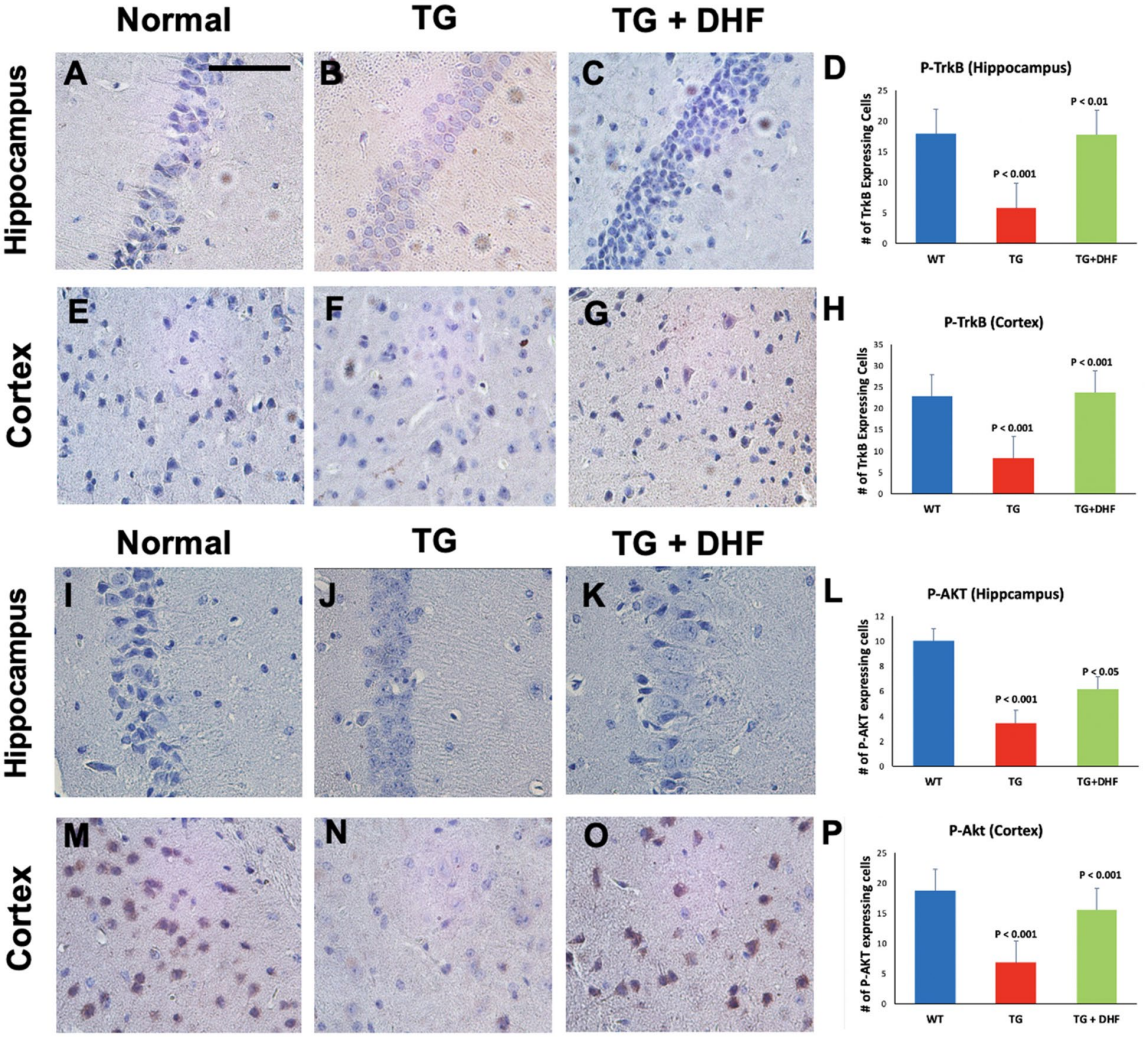
DHF treatment activates the TrkB/Akt pathway. P-TrkB and P-AKT expression in the hippocampus and cortex of Wild Type (WT), Tg26, and DHF treated Tg26 mice (**A**–**P**). Immunohistochemical stained sections show P-TrkB and P-AKT expressing cells in the hippocampal and cortex regions of the mice brains. 3 WT mice, 3 Tg26 mice, and 4 TG + DHF mice were used. A, B, C, E, F, G, I, J, K, M, N, and O are 400 × magnification pictures of the hippocampus and cortex regions of the mice. (**D**) Quantification of P-TRKB expressing cells in the hippocampus: *p* < 0.001, WT versus Tg; *p* < 0.01, Tg versus Tg + DHF. (**H**) Quantification of P-TRKB expressing cells in cortex: *p* < 0.001, WT versus Tg; *p* < 0.001, Tg versus Tg + DHF. One Way ANOVA with Bonferroni's Multiple Comparison post-test. (**L**) Quantification of P-AKT expressing cells in the hippocampus: *p* < 0.001, WT versus Tg; *p* < 0.05, Tg versus Tg + DHF. (**P**) Quantification of P-AKT expressing cells in cortex: *p* < 0.001, WT versus Tg; *p* < 0.001, Tg versus Tg + DHF. The total numbers of hippocampal fields analyzed (WT, Tg26, TG + DHF) for each antibody were P-TrkB (34, 30, 30), P-AKT (45, 45, 60). The total numbers of cortex fields analyzed (WT, Tg26, TG + DHF) for each antibody were P-TrkB (15, 15, 20), P-AKT (45, 45, 60). One Way ANOVA with Bonferroni's Multiple Comparison post-test. scale bar, 100 μm.

To determine the effect of DHF on the phosphorylation of TrkB and downstream signaling pathways in Tg26 mice, we immunohistochemically labeled phosphorylated TrkB (P-TrkB), AKT (P-AKT) were examined in the mouse brains. As shown in Fig. 1A-H, Tg26 mice exhibited significantly decreased phosphorylation of TrkB in comparison to normal mice (*p* < 0.001), and DHF treatment significantly increased the expression of P-TrkB in the hippocampus (*p* < 0.01) and cortex (*p* < 0.001) in comparison to those of the Tg26 mice without DHF treatment. The expression of phospho-AKT (P-AKT) was also decreased significantly in the hippocampus (*p* < 0.001) and cortex (*p* < 0.001) in Tg26 mice, and increased significantly in both the hippocampus (*p* < 0.05) and in the cortex (*p* < 0.001) in the DHF group compared to the Tg26 mice without DHF treatment (Fig. 1I-P).

### DHF downregulates expression of HIV-1 chemokine co-receptors CXCR4 and CCR5

**Figure 2:**
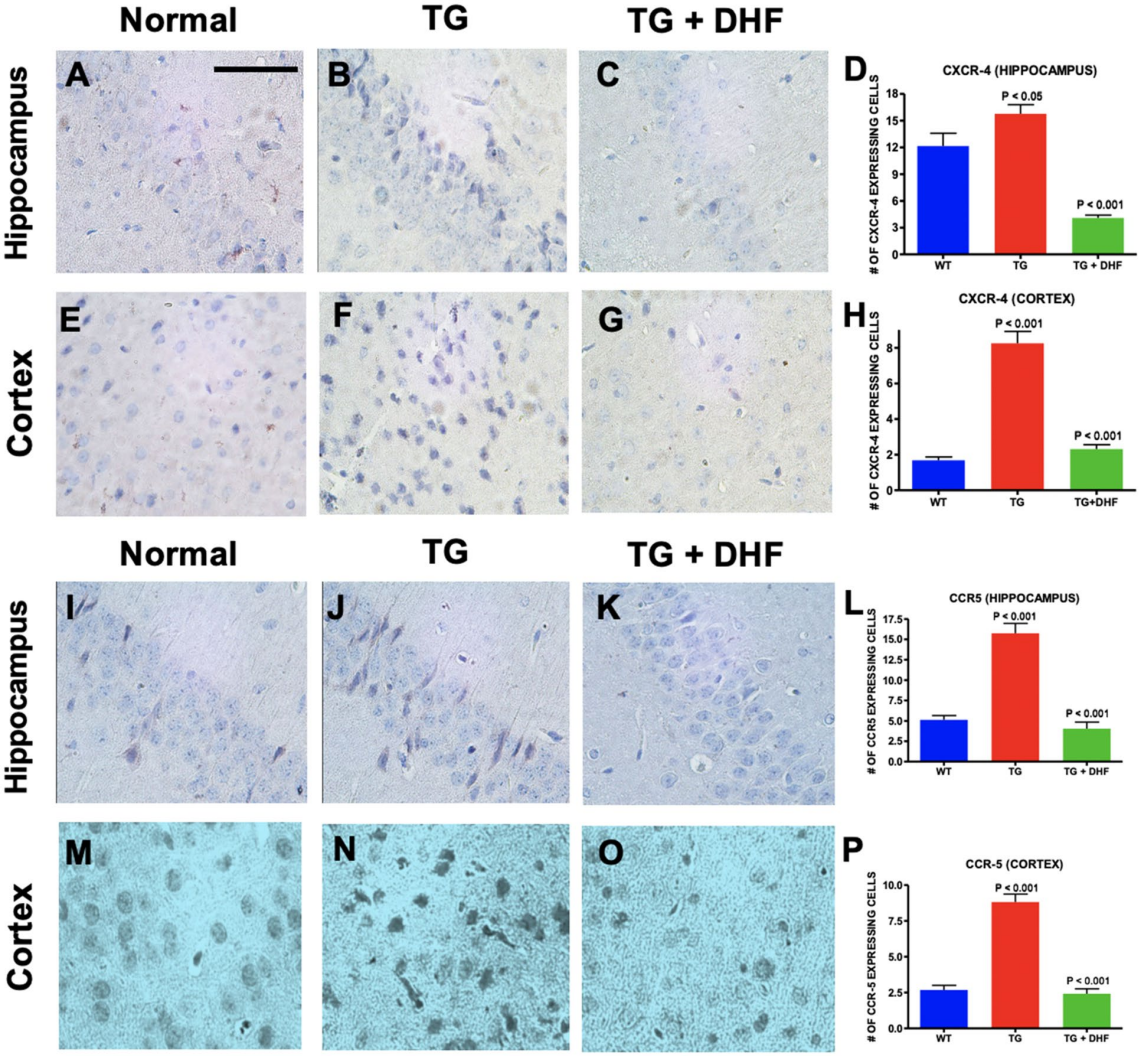
DHF downregulates expression of HIV-1 chemokine co-receptors CXCR4 and CCR5. Legend: CXCR-4 and CCR5 expression in the hippocampus and cortex of Wild Type (WT), Tg26, and DHF treated Tg26 mice (**A**-**P**). Immunohistochemical stained sections show CXCR-4 and CCR5 expressing cells in the hippocampal and cortex regions of the mice brains. 3 WT mice, 3 Tg26 mice, and 4 TG + DHF mice were used. A, B, C, E, F, G, I, J, K, M, N, and O are 400 × magnification pictures of the hippocampus and cortex regions of the mice. (**D**) Quantification of CXCR-4 expressing cells in the hippocampus: *p* < 0.001, WT versus Tg; *p* < 0.05 Tg versus Tg + DHF. (H) Quantification of CXCR-4 expressing cells in cortex: *p* < 0.001, WT versus Tg; *p* < 0.001, Tg versus Tg + DHF. (**L**) Quantification of CCR-5 expressing cells in the hippocampus: *p* < 0.001, WT versus Tg; *p* < 0.001 Tg versus Tg + DHF. (**P**) Quantification of CCR-5 expressing cells in cortex: *p* < 0.01, WT versus Tg; *p* < 0.001, Tg versus Tg + DHF. The total numbers of hippocampal fields analyzed (WT, Tg26, TG + DHF) for each antibody were CXCR-4 (63, 103, 92), CCR5 (110, 92, 71). The total numbers of cortex fields analyzed (WT, Tg26, TG + DHF) for each antibody were CXCR-4 (60, 60, 77), CCR5 (58, 59, 53). One Way ANOVA with Bonferroni's Multiple Comparison post- test. scale bar, 100 μm.

CXCR4 and CCR5 have been implicated in mediating HIV/gp120 neurotoxicity^31,32^. To determine whether DHF mimics the role of BDNF and modulates the availability of chemokine receptors CXCR4 and CCR5 implicated in HIV-1 infection, the levels of CXCR4 and CCR5 expression were examined in the mouse brains by immunohistochemical labeling. We found that in both the hippocampus and cortex of Tg26 mice, CXCR4 (Fig. 2A-H) expression was significantly upregulated in the hippocampus (*p* < 0.05) and cortex (*p* < 0.001) in comparison to normal mice, and DHF treatment significantly downregulated this co-receptor (*p* < 0.001). CCR5 (Fig. 2I-P) expression was also significantly upregulated in the hippocampus (*p* < 0.001) and cortex (*p* < 0.001) in comparison to normal mice, and DHF treatment significantly downregulated CCR5 expression (*p* < 0.001). These results suggest that DHF modulates the expression of CXCR4 and CCR5 on the hippocampal and cortical regions of Tg26 mice.

### DHF treatment downregulates activation of the TLR4 and NFkB

**Figure 3:**
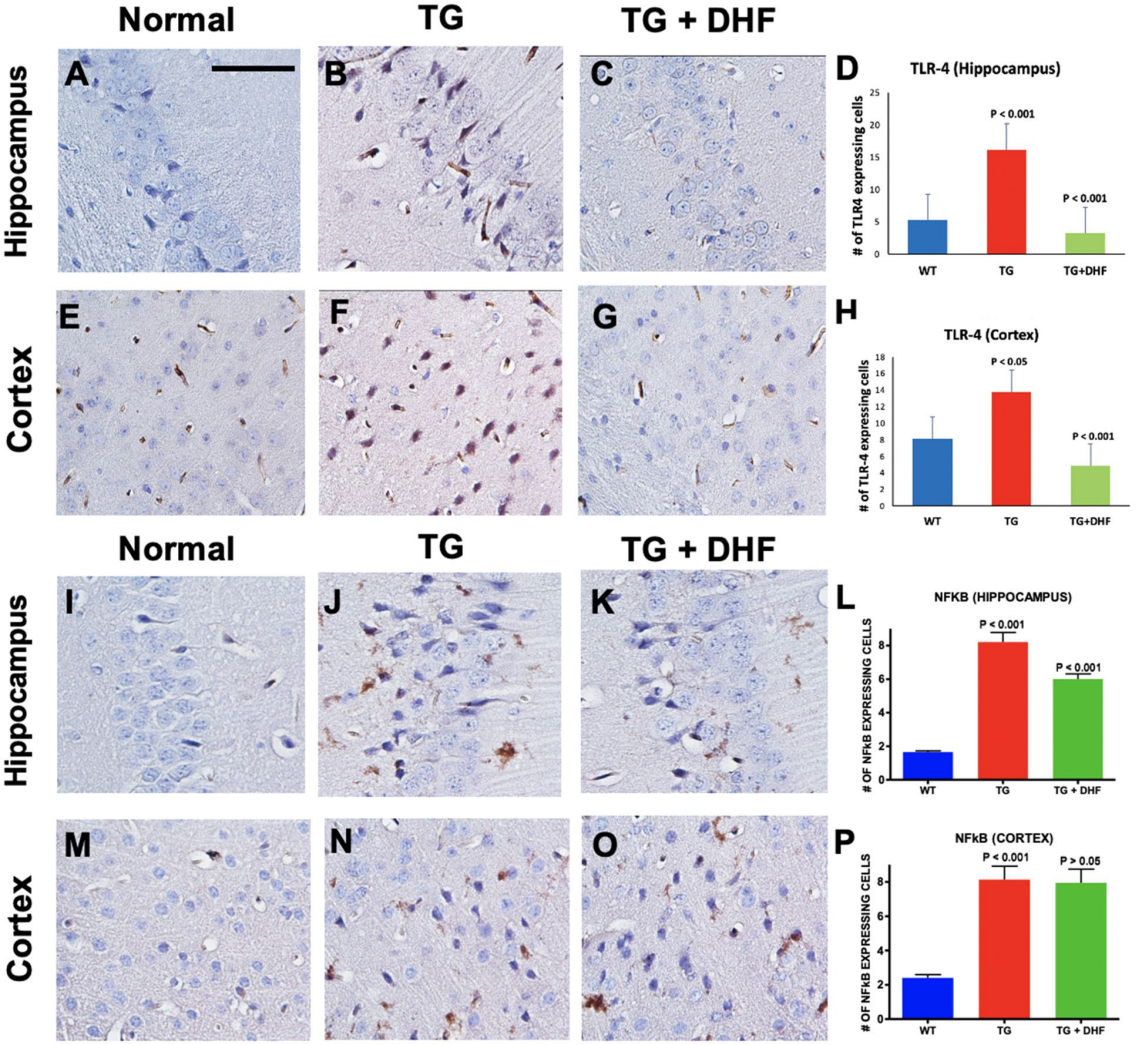
DHF treatment downregulates activation of the TLR4 and NFkB. Legend: TLR4 and NFkB expression in the hippocampus and cortex of Wild Type (WT), Tg26, and DHF treated Tg26 mice (**A**-**P**). Immunohistochemical stained sections show TLR4 and NFkB expressing cells in the hippocampal and cortex regions of the mice brains. 3 and 3 WT mice, respectively, 3 Tg26 mice, and 4 TG + DHF mice were used. A, B, C, E, F, G, I, J, K, M, N, and O are 400 × magnification pictures of the hippocampus and cortex regions of the mice. (**D**) Quantification of TLR4 expressing cells in the hippocampus: *p* < 0.001, WT versus Tg; *p* < 0.001 Tg versus Tg + DHF. (**H**) Quantification of TLR4 expressing cells in cortex: *p* < 0.05, WT versus Tg; *p* < 0.001, Tg versus Tg + DHF. (**L**) Quantification of NFkB expressing cells in the hippocampus: *p* < 0.001, WT versus Tg; *p* < 0.001 Tg versus Tg + DHF. (**P**) Quantification of NFkB expressing cells in cortex: *p* < 0.001, WT versus Tg; *p* > 0.05, Tg versus Tg + DHF. The total numbers of hippocampal fields analyzed (WT, Tg26, TG + DHF) for each antibody were TLR4 (55, 87, 104), NFkB (154, 72, 101). The total numbers of cortex fields analyzed (WT, Tg26, TG + DHF) for each antibody were TLR4 (15, 15, 20), NFkB (15, 15, 20). One Way ANOVA with Bonferroni's Multiple Comparison post- test. scale bar, 100 μm.

The TLR4-NFkB pathway plays a significant role in the activation of proinflammatory responses during infection^33^. Hippocampal and cortical sections were examined immunohistochemically for cells expressing TLR-4 and NF-kB to evaluate the effect of DHF on inflammatory signaling in the brain of Tg26 mice. It was found that the number of cells expressing positive for TLR4 (Fig. 3A-H) and NF-kB (Fig. 3I-P) in Tg26 mice was significantly increased in the hippocampus (*p* < 0.001) and the cortex (*p* < 0.05, *p* < 0.001 respectively). In DHF-treated mice, TLR4 expression was significantly reduced in both regions (*p* < 0.001). For NF-kB however, this effect was only observed in the hippocampus (*p* < 0.001) but not the cortex (*p* > 0.05). These results suggest that in the Tg26 model, the TLR4-NFkB pathway is activated in the hippocampus and brain cortex, and DHF treatment suppresses proinflammatory signaling in these brain regions.

### DHF treatment reduced astrogliosis in the brain of Tg26 mice

**Figure 4:**
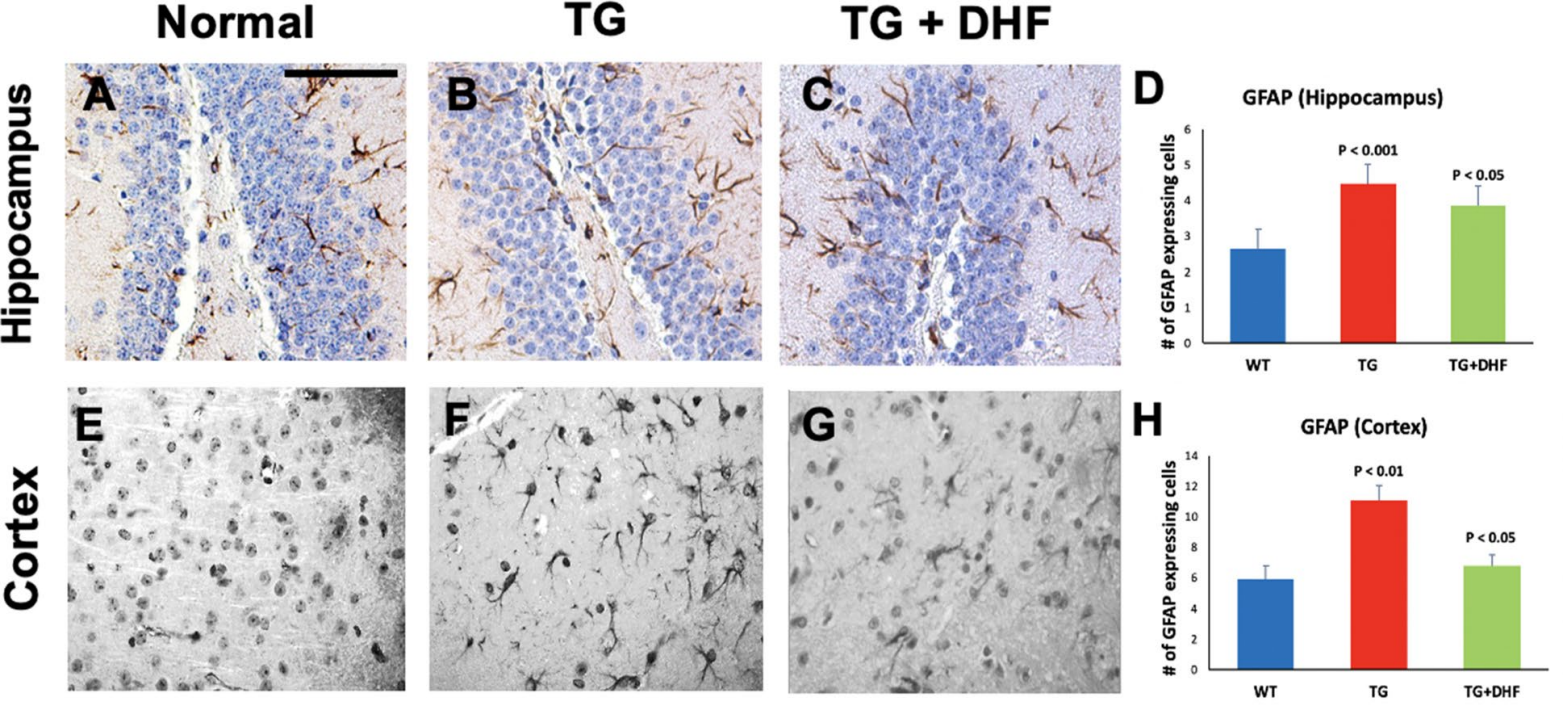
DHF treatment reduced astrogliosis in the brain of Tg26 mice: Legend: GFAP expression in the hippocampus and cortex of Wild Type (WT), Tg26, and DHF treated Tg26 mice (**A**-**H**). Immunohistochemical stained sections show GFAP expressing cells in the hippocampal and cortex regions of the mice brains. 3 WT mice, 3 Tg26 mice, and 4 TG + DHF mice were used. A, B, C and E,F,G are 400 × magnification pictures of the hippocampus and cortex regions, respectively, of the mice. (**D**) Quantification of GFAP expressing cells in the hippocampus: *p* < 0.001, WT versus Tg; *p* < 0.05 Tg versus Tg + DHF. (**H**) Quantification of GFAP expressing cells in cortex: *p* < 0.01, WT versus Tg; *p* < 0.05, Tg versus Tg + DHF. The total numbers of hippocampal fields analyzed (WT, Tg26, TG + DHF) were (61, 74, 107). The total numbers of cortex fields analyzed (WT, Tg26, TG + DHF) were (15, 15, 20). One Way ANOVA with Bonferroni's Multiple Comparison post-test. scale bar, 100 μm.

Reactive astrogliosis is a pathological hallmark of HIV-1 and is apparent in mouse and human HIV + brain tissues, indicated by increased glial fibrillary acidic protein (GFAP) staining^34^. GFAP was significantly increased in the hippocampus (*p* < 0.001) and cortex (*p* < 0.01) in Tg26 mice, indicative of astrogliosis. Interestingly, we found a significant decrease of GFAP (Fig. 4) in the hippocampus (*p* < 0.05) and cortex (*p* < 0.05) of DHF treated Tg26 mice compared to Tg26 mice. These results suggest that DHF reduces astrogliosis in the hippocampus and cortex of Tg26 mice.

### DHF treatment shifts the cytokine profile from Th1 towards Th2 response

**Figure 5:**
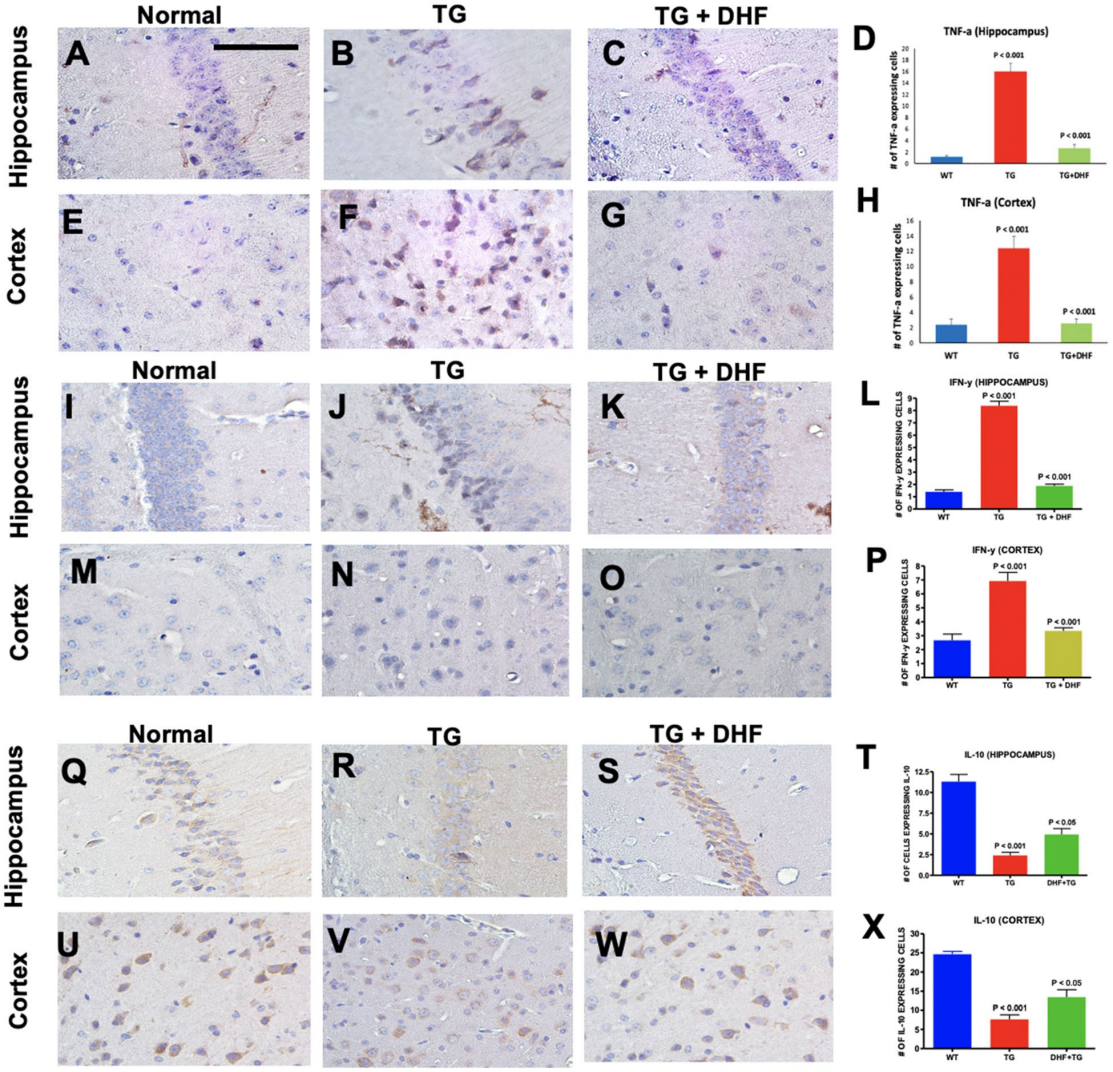
DHF treatment shifts the cytokine profile from Th1 towards Th2 response. Legend: TNF-a, IFN-y and IL-10 expression in the hippocampus and cortex of Wild Type (WT), Tg26, and DHF treated Tg26 mice (**A**–**X**). Immunohistochemical stained sections show TNF-a, IFN-y and IL-10 expressing cells in the hippocampal and cortex regions of the mice brains. 3 WT mice, 3 Tg26 mice, and 4 TG + DHF mice were used for TNF-a. 3 WT mice, 5 Tg26 mice, and 4 TG + DHF mice were used for IFN-y and IL-10. A, B, C, E, F, G, I, J, K, M, N, O, Q, R, S, U, V and W are 400 × magnification pictures of the hippocampus and cortex regions of the mice. (**D**) Quantification of TNF-a expressing cells in the hippocampus: *p* < 0.001, WT versus Tg; *p* < 0.001 Tg versus Tg + DHF. (H) Quantification of TNF-a expressing cells in cortex: *p* < 0.001, WT versus Tg; *p* < 0.001, Tg versus Tg + DHF. (**L**) Quantification of IFN-Y expressing cells in the hippocampus: *p* < 0.001, WT versus Tg; *p* < 0.001, Tg versus Tg + DHF. (**P**) Quantification of IFN-Y expressing cells in cortex: *p* < 0.001, WT versus Tg; *p* < 0.001, Tg versus Tg + DHF. (**T**) Quantification of IL-10 expressing cells in the hippocampus: *p* < 0.001, WT versus Tg; *p* < 0.05, Tg versus Tg + DHF. (X) Quantification of IL-10 expressing cells in cortex: *p* < 0.001, WT versus Tg; *p* < 0.05, Tg versus Tg + DHF. The total numbers of hippocampal fields analyzed (WT, Tg26, TG + DHF) for each antibody were TNF-a (55, 87, 104), IFN-y (154, 72, 101), IL-10 (84, 164, 119). The total numbers of cortex fields analyzed (WT, Tg26, TG + DHF) for each antibody were TNF-a (30, 30, 40), IFN-y (15, 25, 20), IL-10 (10, 18, 19). One Way ANOVA with Bonferroni's Multiple Comparison post- test. scale bar, 100 μm.

Interferon Gamma (IFN-y) and Tumor Necrosis Factor alpha (TNF-a) are proinflammatory Th1/17 cytokines. Interleukin-10 (IL-10), on the other hand, is a Th2 cytokine with potent anti-inflammatory processes. Corresponding to the results described in Sect. 3.4, we observed the same pattern of expression was observed in pro-inflammatory cytokines TNF-a (Fig. 5A-H) and IFN-y (Fig. 5I-P) in both the hippocampus (*p* < 0.001) and cortex (*p* < 0.001). In the Tg26 + DHF group, expression of these cytokines was significantly decreased in both the hippocampus and the cortex (*p* < 0.001). In contrast, expression of anti-inflammatory cytokine IL-10 (Fig. 5Q-X) was significantly reduced in the hippocampus (*p* < 0.001) and the cortex (*p* < 0.001) of Tg26 mice, and DHF significantly reversed this pattern in both the hippocampus (*p* < 0.05) and the cortex (*p* < 0.05). These results suggest that DHF treatment promotes a shift from the Th1/17 towards the Th2 cytokine response in the hippocampus and cortex of Tg26 mice.

### DHF treatment ameliorated mitochondrial dysfunction and biogenesis

**Figure 6:**
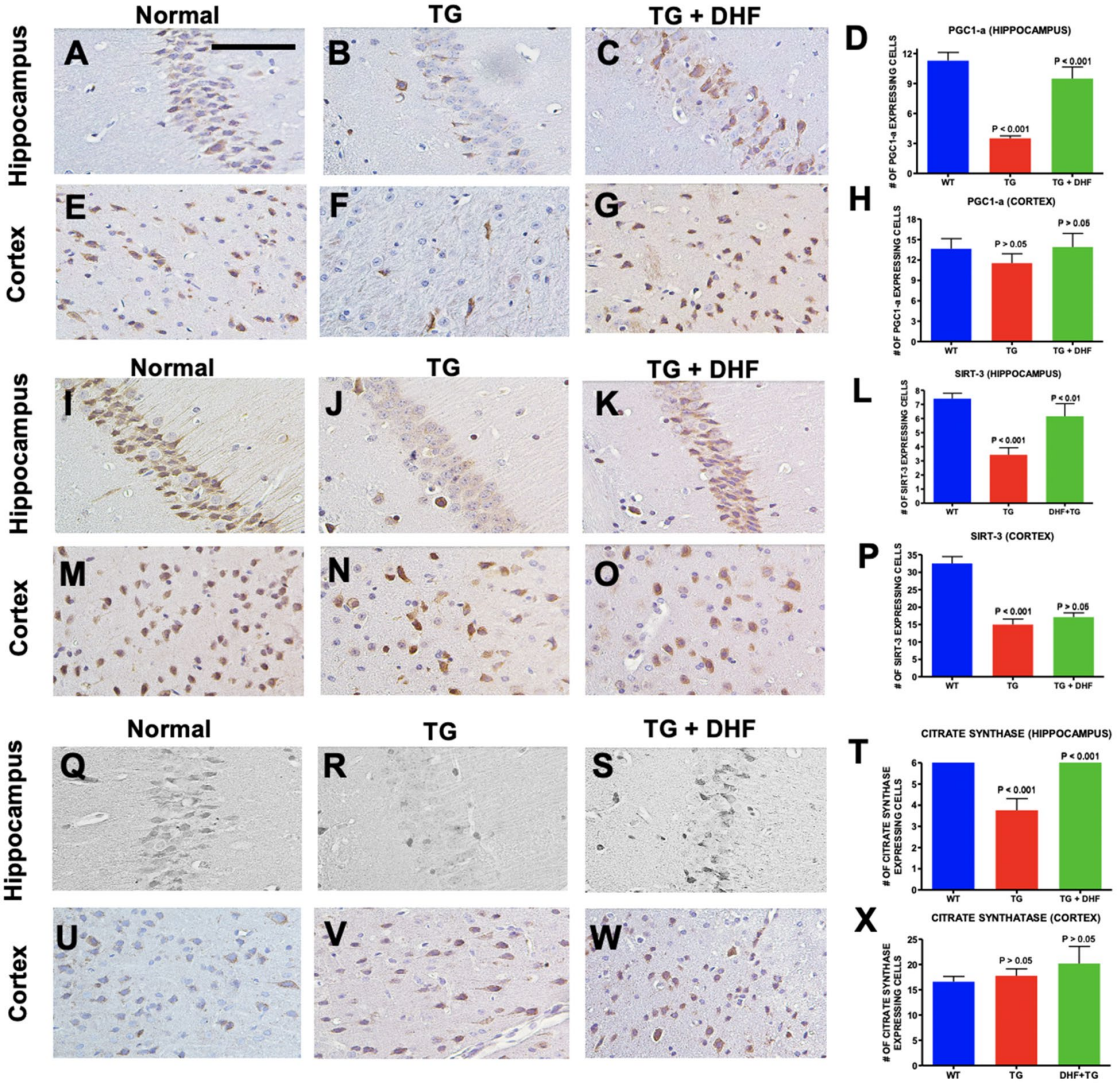
DHF Treatment ameliorated mitochondrial dysfunction and biogenesis. Legend: PGC1a, SIRT-3 and Citrate Synthase expression in the hippocampus and cortex of Wild Type (WT), Tg26, and DHF treated Tg26 mice (**A**–**X**). Immunohistochemical stained sections show PGC1a, SIRT-3 and Citrate Synthase expressing cells in the hippocampal and cortex regions of the mice brains. 4 WT mice, 5 Tg26 mice, and 4 TG + DHF mice were used. A, B, C, E, F, G, I, J, K, M, N, O, Q, R, S, U, V and W are 400 × magnification pictures of the hippocampus and cortex regions of the mice. (**D**) Quantification of PGC1-a expressing cells in the hippocampus: *p* < 0.001, WT versus Tg; *p* < 0.001, Tg versus Tg + DHF. (**H**) Quantification of PGC1-a expressing cells in cortex: *p* > 0.05, WT versus Tg; *p* > 0.05, Tg versus Tg + DHF. (**L**) Quantification of SIRT-3 expressing cells in the hippocampus: *p* < 0.001, WT versus Tg; *p* > 0.01, Tg versus Tg + DHF. (**P**) Quantification of SIRT-3 expressing cells in cortex: *p* < 0.001, WT versus Tg; *p* > 0.05, Tg versus Tg + DHF. (T) Quantification of Citrate Synthase expressing cells in hippocampus: *p* < 0.01, WT versus Tg; *p* < 0.01, Tg versus Tg + DHF. (**X**) Quantification of Citrate Synthase expressing cells in cortex: *p* > 0.05, WT versus Tg; *p* > 0.05, Tg versus Tg + DHF. The total numbers of hippocampal fields analyzed (WT, Tg26, TG + DHF) for each antibody were PGC1a (88, 85, 97), SIRT-3 (109, 134, 94), Citrate Synthase (129, 151, 115). The total numbers of cortex fields analyzed (WT, Tg26, TG + DHF) for each antibody were PGC1a (19, 23, 18), SIRT-3 (20, 25, 19), Citrate Synthase (15, 20, 11). One Way ANOVA with Bonferroni's Multiple Comparison post-test. scale bar, 100 μm.

HIV + brains exposed to cART present altered mitochondrial biogenesis^35^. Hippocampal and cortical regions of Tg26 mice were immunohistochemically stained for Peroxisome proliferator-activated receptor gamma coactivator 1-alpha (PGC-1a) and NAD-dependent deacetylase sirtuin-3 (SIRT-3) to examine changes in mitochondrial biogenesis. Co-transcriptional regulation factor PGC-1a is known to induce mitochondrial biogenesis^36^. We found that PGC1-α (Fig. 6A-H) was significantly decreased (*p* < 0.001) in the hippocampus of Tg26 mice in comparison to wild type mice, and was significantly upregulated (*p* < 0.001) in DHF-treated Tg26 mice in comparison to vehicle-treated Tg26 mice. SIRT3 is localized in mitochondria and is involved in energy metabolism, mitochondrial biogenesis, and mitochondrial fission/fusion^37–40^. Similarly, SIRT3 (Fig. 6I-P) expression was downregulated in the hippocampus of Tg26 mice compared to normal mice (*p* < 0.001) and upregulated in DHF-treated Tg26 mice compared to untreated Tg26 (*p* < 0.01). DHF induced no significant changes in expression of either PGC-1a or SIRT-3 in the cortex of Tg26 mice. These results suggest that DHF improves mitochondrial biogenesis in the hippocampus, but not in the cortex. Citrate synthase is an enzyme-marker for intact mitochondria and is a good indicator of mitochondrial mass^41,42^. We found a significant decrease in the expression of citrate synthase in the hippocampus (*p* < 0.001) of Tg26 mice in comparison to the brains of normal mice, and DHF treatment significantly increased its expression in the hippocampus (*p* < 0.001) (Fig. 6Q-X). However, we observed no significant changes in citrate synthase levels between all three groups in the cortex (*p* > 0.05).

### DHF treatment improved mitochondrial fission, but not fusion

**Figure 7:**
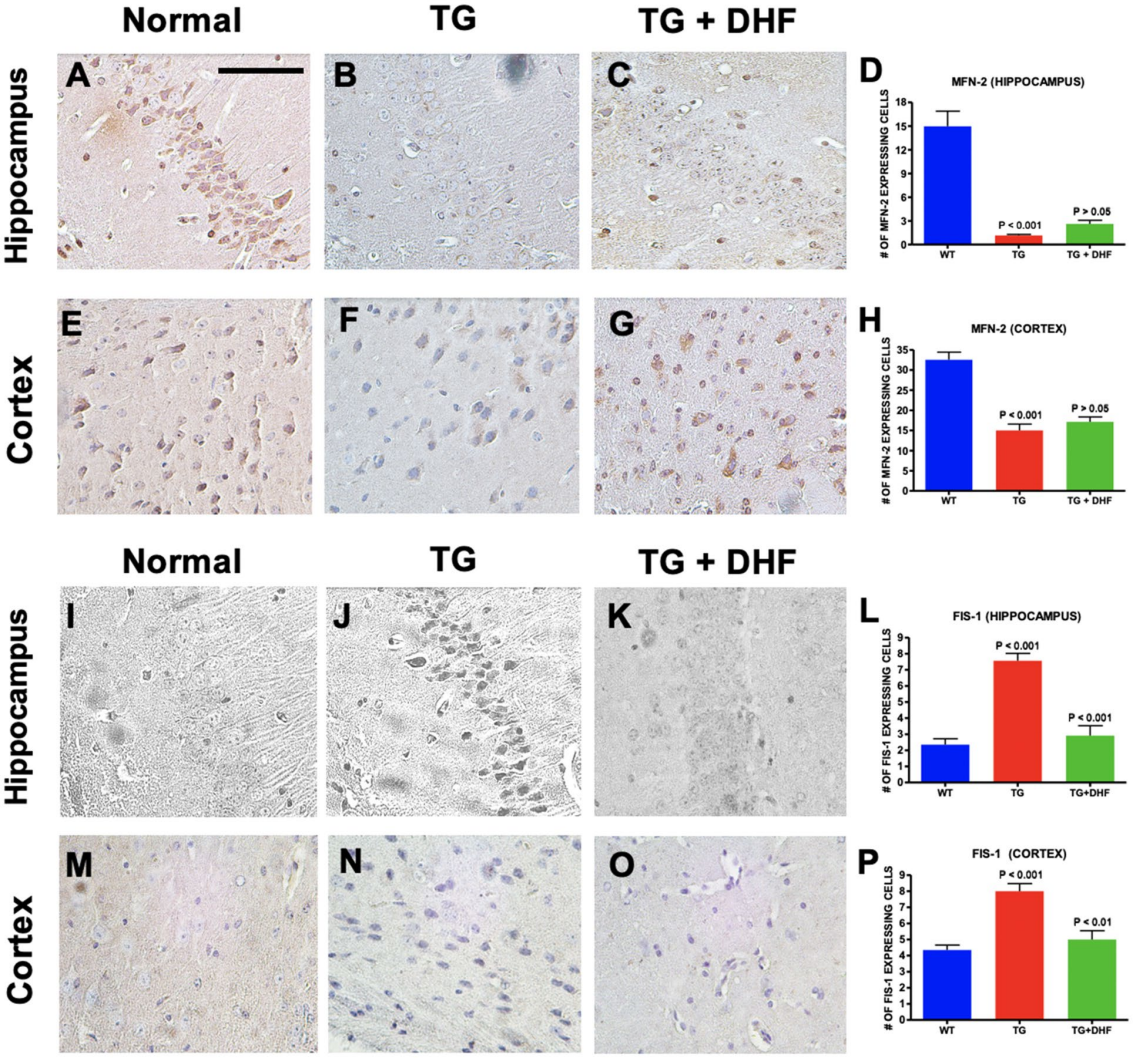
DHF treatment improved mitochondrial fission, but not fusion. Legend: MFN-2 and FIS-1 expression in the hippocampus and cortex of Wild Type (WT), Tg26, and DHF treated Tg26 mice (**A**-**P**). Immunohistochemical stained sections show MFN-2 and FIS-1 expressing cells in the hippocampal and cortex regions of the mice brains. 4 WT mice, 5 Tg26 mice, and 4 TG + DHF mice were used for MFN-2. 4 WT mice, 3 Tg26 mice, and 3 Tg + DHF mice were used for FIS-1. A, B, C, E, F, G, I, J, K, M, N, and O are 400 × magnification pictures of the hippocampus and cortex regions of the mice. (**D**) Quantification of MFN-2 expressing cells in the hippocampus: *p* < 0.001, WT versus Tg; *p* > 0.05, Tg versus Tg + DHF. (**H**) Quantification of MFN-2 expressing cells in cortex: *p* < 0.001, WT versus Tg; *p* > 0.05, Tg versus Tg + DHF. (**L**) Quantification of FIS-1 expressing cells in the hippocampus: *p* < 0.001, WT versus Tg; *p* < 0.001, Tg versus Tg + DHF. (**P**) Quantification of FIS-1 expressing cells in cortex: *p* < 0.001, WT versus Tg; *p* < 0.01, Tg versus Tg + DHF. The total numbers of hippocampal fields analyzed (WT, Tg26, TG + DHF) for each antibody were MFN-2 (103, 93, 63), FIS-1 (40, 30, 33). The total numbers of cortex fields analyzed (WT, Tg26, TG + DHF) for each antibody were MFN-2 (17, 22, 18), FIS-1 (20, 20, 15). One Way ANOVA with Bonferroni's Multiple Comparison post-test. scale bar, 100 μm.

Mitochondrial fission/fusion are simultaneous actions that occur in mitochondrial structures to regulate their morphology^43^. To test if DHF had an effect on mitochondrial fission/fusion, sections of the hippocampus and cortex of Tg26 mice were immunohistochemically labeled for mitochondrial fusion-2 (MFN-2) and mitochondrial fission (FIS-1). MFN-2 plays a role in the regulation of fusion processes^44^. While MFN-2 (Fig. 7A-H) was significantly downregulated in both the hippocampus and cortex of Tg26 mice (*p* < 0.001), no significant increase was observed in the DHF treated group (*p* > 0.05). On the other hand, we found a significant increase in the expression of FIS-1, which is involved in the fragmentation of mitochondrial networks, (Fig. 7I-P) in both the hippocampus (*p* < 0.001) and cortex (*p* < 0.001) of Tg26 mice in comparison to wild type mice. Interestingly, DHF significantly lowered the expression of FIS-1 in Tg26 mice close to normal levels in the hippocampus (*p* < 0.001) and the cortex (*p* < 0.01). These results suggest that DHF may play a role in restoring normal mitochondrial fusion and fission in the hippocampus and cortex of Tg26 mice, but more research is necessary to reach a firm conclusion.

### DHF treatment did not improve metabolic function and ER-mitochondria communication

**Figure 8:**
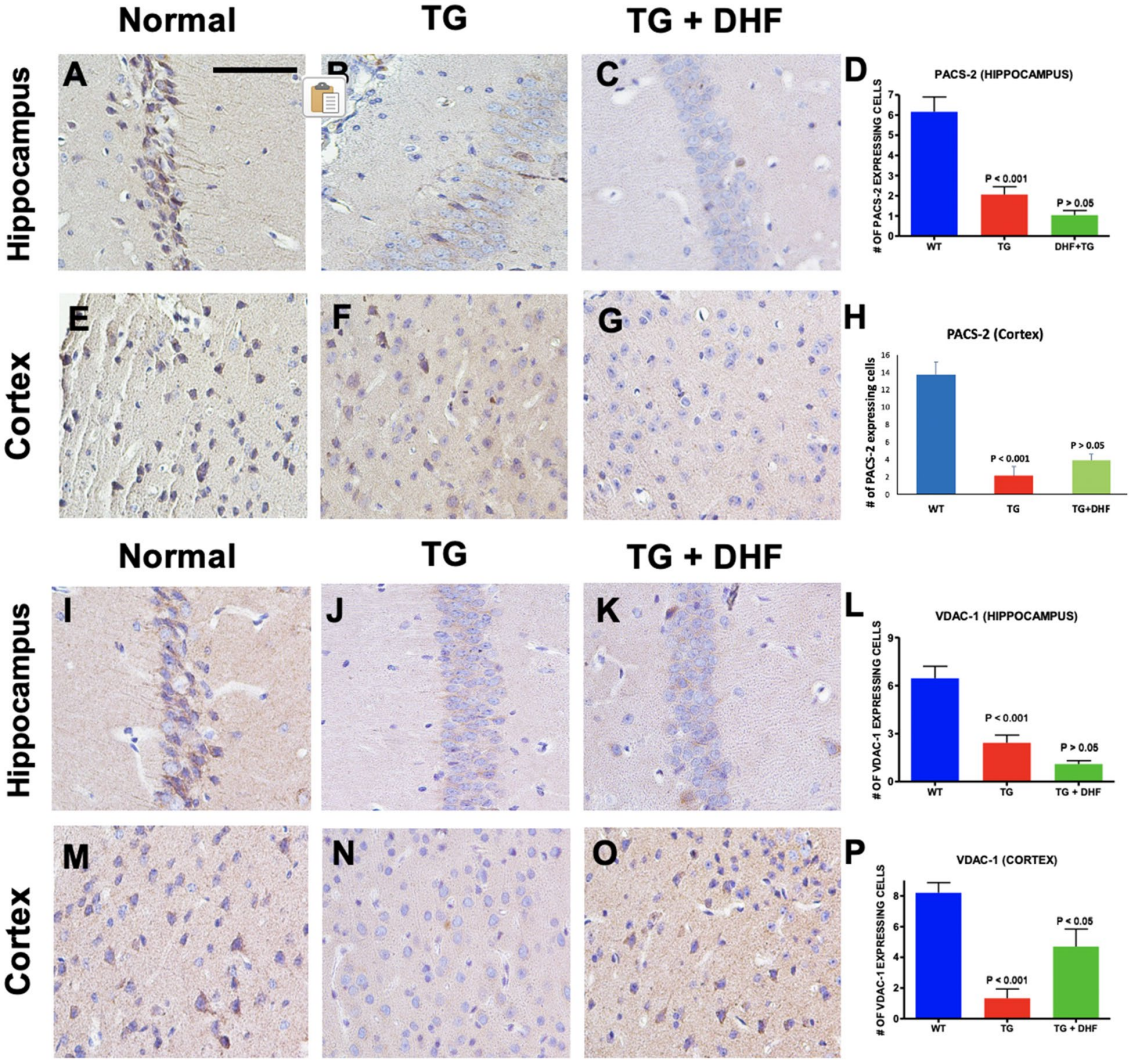
DHF treatment did not improve metabolic function and ER-mitochondria communication. Legend: PACS-2 and VDAC-1 expression in the hippocampus and cortex of Wild Type (WT), Tg26, and DHF treated Tg26 mice (**A**-**P**). Immunohistochemical stained sections show PACS-2 and VDAC-1 expressing cells in the hippocampal and cortex regions of the mice brains. 3 WT mice, 3 Tg26 mice, and 4 TG + DHF mice were used. A, B, C, E, F, G, I, J, K, M, N, and O are 400 × magnification pictures of the hippocampus and cortex regions of the mice. (**D**) Quantification of PACS-2 expressing cells in the hippocampus: *p* < 0.001, WT versus Tg; *p* > 0.05, Tg versus Tg + DHF. (**H**) Quantification of PACS-2 expressing cells in cortex: *p* < 0.001, WT versus Tg; *p* > 0.05, Tg versus Tg + DHF. (**L**) Quantification of VDAC-1 expressing cells in the hippocampus: *p* < 0.001, WT versus Tg; *p* > 0.05, Tg versus Tg + DHF. (**P**) Quantification of VDAC-1 expressing cells in cortex: *p* < 0.001, WT versus Tg; *p* < 0.05, Tg versus Tg + DHF. The total numbers of hippocampal fields analyzed (WT, Tg26, TG + DHF) for each antibody were PACS-2 (62, 110, 112), VDAC-1 (64, 119, 124). The total numbers of cortex fields analyzed (WT, Tg26, TG + DHF) for each antibody were PACS-2 (15, 10, 20), VDAC-1 (15, 15, 20). One Way ANOVA with a Bonferroni's Multiple Comparison post-test. Scale bar, 100 μm.

To determine the effect of DHF on mitochondrial metabolic function and ER-mitochondria communication, we examined changes in expression of Phosphofurin Acidic Cluster Sorting Protein 2 (PACS-2) (Fig. 8A-H) and Voltage Dependent Anion Channel 1 (VDAC-1) (Fig. 8I-P) by using immunohistochemical labeling. PACS-2 is a multifunctional sorting protein that regulates communication between the mitochondria and the endoplasmic reticulum^45^. In Tg26 mice, it was found that PACS-2 was significantly downregulated in the hippocampus (*p* < 0.001) and the cortex (*p* < 0.001). In the DHF treated group, no significant changes were found in either the hippocampus (*p* > 0.05) or the cortex (*p* > 0.05). VDAC-1 is an outer mitochondrial membrane and plasma membrane channel that regulates the release of extracellular ATP^46^. VDAC-1 was also significantly decreased in the hippocampus (*p* < 0.001) and cortex (*p* < 0.001) of Tg26 mice, but DHF only had a positive effect in the cortex (*p* < 0.05). These results suggest that DHF does not have a significant effect in restoring mitochondrial-ER axis homeostasis.

## Discussion

In this study, we demonstrated for the first time that administration of DHF resulted in reduced expression of HIV-1 chemokine co-receptors CXCR4 and CCR5, decreased astrogliosis, suppressed inflammatory activity, and improved mitochondrial function/biogenesis in the hippocampal and brain cortical regions of Tg26 mice. DHF also enhanced the phosphorylation of TrkB and its downstream signaling pathway Akt, which was correlated with a downregulation in downstream NFkB signaling, indicating that enhancing TrkB activation is a potential therapeutic mechanism in HAND.

TrkB is a receptor of BDNF that plays a role in stimulating neuronal survival, morphogenesis, and plasticity^47^, and DHF upregulates TrkB phosphorylation via the Akt pathway^48^. The PI3K/Akt signaling pathway protects against apoptosis and promotes neuronal survival^48^. DHF activates the TrkB receptor and the PI3K/AKT and MAPK pathway in hippocampal neurons^49^. In the Tg26 model, immunohistochemical staining for phosphorylated TrkB and Akt (Fig. 1) in the hippocampus and cortex revealed upregulated phosphorylation of the TrkB/Akt pathway in DHF-treated Tg26 in comparison to Tg26 mice without treatment. These results indicate that DHF successfully crossed the BBB and sufficiently binded to the TrkB receptor. NFkB, which is downstream of Akt^50^, was upregulated in the same brain regions of Tg26 mice, and DHF reversed this change (Fig. 3A-H). These findings suggest that the antiinflammatory neuroprotective effects of DHF may be via activation of the TrkB/Akt pathway and downregulation of downstream NF-kB.

Several studies have demonstrated that astrocytes, neurons, and microglia express chemokine receptors CXCR4 and CCR5 which facilitate HIV-1 entry, making them highly susceptible to cytokine signaling and HIV-1 induced neurotoxicity^32,34,51–53^. We demonstrate that CXCR4 and CCR5 immunoreactivity were significantly upregulated in Tg26 hippocampi and cortices as expected in a HAND model. BDNF, by activating TrkB, has been shown to be neuroprotective *in vivo*^10^ and *in vitro*^11^ against two strains of gp120, which binds to CXCR4 or CCR5^11,53,54^. In the hippocampus and brain cortex of BDNF (+ /-) mice, it was found previously that CXCR4 is highly expressed particularly in neurons rather than astrocytes^53^, perhaps because neurons are known to express full length TrkB receptors^56^. This suggests that BDNF may not regulate CXCR4 expression in nonneuronal cells. Nevertheless, the BDNF-TrkB pathway’s ability to modulate CXCR4 expression underlies BDNF’s neuroprotective properties against gp120, and our findings show that DHF successfully downregulates CXCR4 expression. The cognitive deficits induced by HIV-1 may also be due in part to the overexpression of CCR5; neuronal overexpression of CCR5 causes memory deficits, and decreasing the function of CCR5 improved long-term potentiation and neuroplasticity^56^. In a transgenic model of HIV-associated brain injury induced by a CXCR4-utilizing viral envelope gp120, CCR5 knockout prevented neuronal injury and behavioral impairment^57^. PWH carry a homozygous deletion of 32 base pairs in the CCR5 gene, which prevents CCR5 cell surface expression and protects against infection by HIV-1 R5-tropic strains, exhibit less cognitive impairment^58–60^. Furthermore, CCR5 antagonist Maraviroc improves neurocognitive status in PWH when administered as a supplement to cART regimens^61^. Our findings suggest that DHF enhances neuroprotection and mediates neurotoxicity due to HIV-1 associated genes by reducing CXCR4 and CCR5 expression in the hippocampus and brain cortex (Fig. 2), perhaps by increasing BDNF-TrkB signaling mechanisms, in the Tg26 model. Downregulating NF-kB may also have attenuated the expression of CXCR4 and CCR5, as these chemokine receptors play a role during neuroinflammation in HAND and previous studies suggest that NFkB mediates their expression^62–64^. Given that TrkB activation modulates CXCR4 and CCR5 expression, our findings suggest that this effect may be mediated by NF-kB^54,65^.

Viral proteins such as gp120, Tat, and Vpr are indirectly neurotoxic by binding to CXCR4 and CCR5 and activating macrophages, microglia, and astrocytes^66^. This indirect neurotoxicity fosters an environment of chronic inflammation that is characteristic of HAND^66^. HAND is characterized by glial activation, cytokine/chemokine dysregulation, and neuronal damage and loss^9,67^. Even in the cART era, microgliosis, microglial nodules, astrocytosis, and other neuroinflammation indicators have been found in the postmortem brains of PWH^9,68,69^, especially in the memory-associated areas of the brain including the hippocampus and entorhinal and temporal cortices^69^. A major barrier in the treatment of HIV-1 is the reduced activity of cART in the brain, where long-lived macrophages/microglia and astrocytes serve as viral reservoirs^6,7^. HIV-1 Tg26 mice exhibited an increase of GFAP + astrocytes in the cerebral cortex and hippocampus compared to WT mice, and DHF reversed these changes (Fig. 4). Various mouse models of HAND and human HIV + brain tissues have indicated that reactive astrogliosis is a pathological hallmark of HIV-1^34,70^. It is suggested that additional treatment targeting astrocytosis may be necessary to further reduce the effects of HIV-1 in the CNS^71^. Vartak-Sharma et al. demonstrated that astrogliosis in HIV-associated neuroinflammation may be mediated by NFkB signaling via astrocyte elevated gene-1^72,73^. Our findings suggest that DHF protects against HIV-mediated astrogliosis, which may be through down-regulating NF-kB activation^74,75^. This is consistent with a study that examined the effect of DHF in mice exposed to perinatal hypoxia and ischemia^76^.

In our findings, we show that DHF treatment mitigates neuroinflammation in the hippocampus and cortex of TG26 mice (Figs. 3–5). Previously, it has been reported that TNF, IL-1β and IFN-γ are produced by activated monocytes/macrophages, microglia and T cells of PWH presenting signs of dementia, and the cerebrospinal fluid of PWH contain higher levels of proinflammatory cytokines such as IFN-γ, TNF-α, IL-2, IL-6, IL-7, and IL-8^77^. TNF-α is recognized to be an inducer of neuronal injury as it increases the permeability of the BBB, resulting in the migration of HIV-infected monocytes into the CNS^78^. IFN-γ correlated with the severity of neurologic impairment in PWH^79^. Prolonged abnormal presence of IFN-γ reduced heme oxygenase-1 expression in human astrocytes, which contributes to oxidative stress, another pathogenic characteristic of HAND^80^. Several previous studies have found that HIV viral genes induce the expression of IL-10; however, in the Tg26 mouse model^81–85^, we found the opposite. DHF upregulated IL-10, similar to our previous findings in a murine model for multiple sclerosis, a neurodegenerative disease^25^. DHF likely downregulated TNF-a indirectly by activating the TrkB signaling cascade, which in turn down-regulated NFkB and suppressed the neuroinflammatory response. The reduced levels of TNF-a may lead to increased levels of BDNF because TNF-a is known to prevent the activity of the glucocorticoid receptor, forming a positive-feedback type mechanism^84^.

TLR-4 is an upstream mediator of NF-kB and is upregulated in astrocytes during HIV-1 infection^85^. The HIV-1 Tat protein binds to the TLR4-MD2-CD14 complex, activating the NF-kB pathway, and, in turn, inducing the production of pro-inflammatory cytokines^86,87^. In our findings with Tg26 mice, TLR4-NFkB activation possibly triggered the secretion of proinflammatory cytokines TNF-a and IFN-y, and inhibited secretion of anti-inflammatory IL-10. It is widely accepted that NFkB is activated during HIV-induced neuroinflammation^88,89^. The activation of NFkB often acts as an initiating signal for the transcription of HIV-1, and targeting this signaling pathway could prevent ongoing low-level neurodegeneration by disturbing HIV-1’s ability to sense immune cell activation^90^. Inhibition of NF-κB activity can reverse neuronal autophagy induced by Tat^91^. We recently reported that HIV-1 Vpr-induced proinflammatory response and apoptotic cell death are mediated through the NF-kB activation in astrocytes^92^. Targeting NFkB may ameliorate HIV-associated neuroinflammation. We report for the first time that DHF downregulates TLR4-NFkB signaling during HIV-associated neuroinflammation, resulting in an anti-inflammatory shift in cytokine release. Previously Park et al.^93^ reported that DHF reduces LPS-induced NF-kB activity via the suppression of the nuclear translocation of NF-kB p65 and the degradation of inhibitor kB and reduced inflammation (70). Our data sheds light on DHF’s ability to reduce neuroinflammation by attenuating the proinflammatory responses.

Persistent inflammation can be attributed to the metabolic abnormalities in PWH^94,97^. HIV-1 transgenic mice exhibit mitochondrial abnormalities associated with impaired energy homeostasis^96^. Impaired mitochondrial metabolism, altered mitochondrial biogenesis, and abnormal mitochondrial morphology are prominent in postmortem brains of PWH^97–100^. Our findings with SIRT3 and citrate synthase (Fig. 6I-X) suggest that DHF improves mitochondrial biogenesis and function. SIRT3 mediated mitochondrial biogenesis is regulated by PGC-1α^101^. Just as we found in the Tg26 model, PGC-1a levels are reduced in HIV + brains exposed to ART^35^. In a TBI model, it was previously established that DHF restored levels of PGC-1a^102^. This mechanism, similar to that of what we present in our animal model of HAND (Fig. 6 A-H), is likely TrkB dependent, as the BDNF receptor activates the cAMP-response-element-binding protein (CREB), a transcription factor that regulates PGC-1a^102^. Low PGC-1α levels are associated with stimulating NF-kB activation, as seen in our Tg26 model, suggesting a link between metabolic disturbances and the inflammatory response. DHF appears to ameliorate these changes. DHF has also been shown to exert neuroprotection independently through its antioxidant properties^103,104^, and SIRT3 regulates antioxidant activity^105^, so DHF may perhaps exert antioxidant effects in Tg26 mice. Mitochondrial dynamics also play an important role in HAND^106^.

In current literature, there are mixed results as to how the HIV alters mitochondrial fusion/fission. While one study determined that HIV-1 Vpr post-transcriptionally reduces the expression of MFN-2 and causes a loss in mitochondrial membrane potential^107^, another study claims that MFN-2 levels remained unchanged after exposure to HIV-1 tat in neurons. Interestingly, however, another study presented that in the brains of HIV + donors, mitochondrial fusion protein MFN-1 expression was increased in neurons, suggesting a shift towards mitochondrial fusion. For the first time, we show that MFN-2 levels decrease in the Tg26 model, and DHF did not affect MFN-2 levels in this model (Fig. 7A-H). As for mitochondrial fission, one study presented that HIV proteins induce the translocation of fission protein DRP-1 to promote mitochondrial fission^108^. Another study, however, determined that FIS-1 immunoreactivity was decreased in gp120 Tg mice^99^. In the Tg26 model, it was seen that mitochondrial fission was abnormally increased in the hippocampus and cortex regions by analysis of FIS-1, and unlike MFN-2, DHF did have a significant restoring effect on FIS-1 (Fig. 7I-P), suggesting that DHF may have an ameliorative effect on mitochondrial fission. Mitochondrial fission/fusion is also regulated by NF-kB, suggesting an intricate relationship between mitochondrial dysfunction and chronic inflammation in HAND^109^. Together, our data suggests that HIV-1 viral proteins (expressed in Tg26 mice) produce chronically dysfunctional mitochondria, possibly contributing to the HAND pathology. DHF treatment may increase mitochondrial integrity, function by perhaps downregulating mitochondrial fission. Additionally, mitochondrial dysfunction and ER stress are pathological characteristics of HAND, which results in the disruption of ER-mitochondria communication^110–113^. In turn, this may lead to disturbances in mitochondrial bioenergetics/dynamics. For the first time, we show that PACS-2 (Fig. 8A-H) and VDAC-1 (Fig. 8I-P), both of which are involved in ER-mitochondria communication, mitophagy, and calcium influx^114,115^, are dysregulated in the Tg26 hippocampus and cortex. However, we did not observe a significant effect of DHF treatment on PACS-2 and VDAC-1 indicating need for further research to determine the effect of DHF on ER-mitochondria communication.

While the study lays a strong foundation for the potential therapeutic efficacy of DHF, we also highlight several limitations. Our study only used in-vivo studies and IHC methods, so further research is needed to confirm the mechanisms underlying DHF’s neuroprotective effect in HAND. Future studies should also use various techniques such as western blots and RT-PCR to further analyze protein levels and interactions with and without administration of DHF. Future research should also specifically explore if the shift towards anti-inflammation and metabolic homeostasis is independent or dependent of the TrkB-Akt signaling cascade. Behavioral testing and neuronal functional assays will also better evaluate the role of DHF in mediating the cognitive deficits of HAND. Furthermore, while the Tg26 mouse model has been used to study various HIV-associated comorbidities, its potential use for HAND has not been extensively investigated. Future studies should investigate DHF in other animal models such as HIV-1 humanized mice. By addressing these limitations with future research, we can gain better insight into DHF’s potential as an adjunct therapeutic agent to current antiviral therapy.

## Conclusion

We investigated the neuroprotective effects of BDNF using 7,8-dihydroxyflavone, a small molecule that is a bioactive high-affinity TrkB agonist utilizing the HIV-1 transgenic mouse model (Tg26). Our in vivo studies identified that HIV-1 Tg26 mice have neurologic deficits, associated with hippocampal and brain cortical changes in astrogliosis, CXCR4/CCR5 expression, inflammatory activity, and mitochondrial changes, all of which are characteristic of HAND. Following treatment with DHF in Tg26 mice, the mice exhibited a reversal of the pathological changes, suggesting the therapeutic potential of DHF in HAND. We provide an overview of how targeting BDNF-TrkB signaling in the pathophysiology of HAND may be relevant for future therapies, and how 7,8 Dihydroxyflavone may be a potential adjunct therapeutic agent to current antiviral therapy.

## Data Availability

"The datasets supporting the conclusions of this article are available in the National Addiction and HIV Data Archive Program repository, NAHDAP-122302.

## Abbreviations

cART: Combined antiretroviral therapy
PWH: People with HIV
HAND: HIV
CNS: Central nervous system
BDNF: Brain derived neurotrophic factor
TrkB: Tropomyosin-receptor-kinase B
HAD: HIV-associated dementia
BBB: Blood-brain barrier
DHF: 7,8-Dihydroxyflavone
WT: Wild-type
CXCR4: C-X-C chemokine receptor type 4
CCR5: C-C chemokine receptor type 5
Tg26: Transgenic mice
DMSO: Dimethyl sulfoxide
PBS: Phosphate-buffered saline
GFAP: Glial fibrillary acidic protein
P-TRKB: Phospho-tropomyosin receptor kinase B
P-AKT: Phospho-protein kinase B
IFN-y: Interferon-gamma
TNF-a: Tumor necrosis factor alpha
IL-10: Interleukin-10
NFkB: Nuclear factor kappa-light-chain-enhancer of activated B cells
TLR-4: Toll-like receptor 4
SIRT-3: Sirtuin 3
PGC1a: Peroxisome proliferator-activated receptor gamma coactivator 1 α
MFN-2: Mitofusin 2
FIS-1: Mitochondrial fission-1 protein
PACS2: Phosphofurin Acidic Cluster Sorting Protein 2
VDAC-1: Voltage Dependent Anion Channel 1
