# Author Correction: 7,8-Dihydroxyflavone improves neuropathological changes in the brain of Tg26 mice, a model for HIV-associated neurocognitive disorder

**DOI:** 10.1038/s41598-021-99820-w

**Published:** 2021-10-06

**Authors:** Joseph Bryant, Sanketh Andhavarapu, Christopher Bever, Poornachander Guda, Akhil Katuri, Udit Gupta, Muhammed Arvas, Girma Asemu, Alonso Heredia, Volodymyr Gerzanich, J. Marc Simard, Tapas Kumar Makar

**Affiliations:** 1 Institute of Human Virology, Baltimore, MD 21201 USA; 2 Research Service, Veterans Affairs Center, Baltimore, MD 21201 USA; 3 Department of Neurosurgery, University of Maryland, Baltimore, MD 21201 USA

Correction to: *Scientific Reports* 10.1038/s41598-021-97220-8, published online 16 September 2021

The original version of this Article contained an error in the spelling of the author J. Marc Simard which was incorrectly given as Marc J. Simard. The original Article has been corrected.

